# Supplementary material for: Association of Telomere Length in T Lymphocytes, B Lymphocytes, NK Cells and Monocytes with Different Forms of Age-Related Macular Degeneration
Source: Biomedicines. 2024 Aug 19;12(8):1893. doi: 10.3390/biomedicines12081893 (PMC11351114; doi:10.3390/biomedicines12081893)
Supplement: Supplementary file 1 [file biomedicines-12-01893-s001.zip › biomedicines-3133996-supplementary.pdf]

Supplementary file

Association of Telomere Length in T lymphocytes, B lymphocytes, NK cells and Monocytes with Different Forms of Age-Related Macular Degeneration

Anait S. Khalatyan [1*], Anastasiya N. Shishparenok [2], Konstantin S. Avetisov [1], Yulia A. Gladilina [2], Varvara G. Blinova [2] and Dmitry D. Zhdanov [2,*]

[1] Krasnov Research Institute of Eye Diseases, 11A, B, Rossolimo str., Moscow, 119021, Russian Federation; anaits92@gmail.com (A.S.K.); avetisovks@gmail.com (K.S.A.);

[2] Laboratory of Medical Biotechnology, Institute of Biomedical Chemistry, 10/8 Pogodinskaya St., 119121 Moscow, Russia; a.shishparyonok@ibmc.msk.ru (A.N.S.); gladilinaya@ibmc.msk.ru (Y.A.G.);

* Corresponding author at: Krasnov Research Institute of Eye Diseases, 11A, B, Rossolimo str., Moscow, 119021, Russian Federation Tel. +79586352034; Email: anaits92@gmail.com

Abstract

Background: Age plays a primary role in the development of age-related macular degeneration (AMD). Telomere length (TL) is one of the most relevant biomarkers of aging. In our study, we aimed to determine the association of TL of T lymphocytes, B lymphocytes, NK cells or monocytes with different forms of AMD.

Methods: Our study included 62 patients with AMD: geographic atrophy (GA), neovascular AMD (NVAMD) with and without macular atrophy, and 22 healthy controls. Each leukocyte subtype was isolated from peripheral blood by immunomagnetic separation and DNA was purified. TL in genomic DNA was determined using qPCR by amplifying the telomere region with specific oligonucleotide primers and normalizing to the control gene. Statistical analysis was performed using R version 4.5.1.

Results: We observed a statistically significant increase in TL in T cells between the control and NVAMD groups, but not for the GA group. B cells and monocytes showed a significant decrease in TL in all AMD groups. TL in NK cells were not decreased in all the AMD groups. TL in monocytes had the strongest association with AMD. It reflects a person's "telomeric status" and may become a diagnostic hallmark of these degenerative process.

Key words: age-related macular degeneration; macular atrophy; telomere length; lymphocytes; monocytes.

Table S1. Comparison of telomere lengths across leukocytes subpopulations from control group of patients

Leukocytes	B cells	Monocytes	NK cells
T cells	$p < 0.001$	$p < 0.001$	$p = 0.006$
B cells		$p = 0.302$	$p < 0.001$
Monocytes			$p < 0.001$

Table S2. p-values for the paired comparisons of TL in T cells between the groups

Group	NVAMD MA	NVAMD no MA	GA
Controls	$p < 0.001$	$p < 0.001$	$p = 0.107$
NVAMD MA		$p = 0.401$	$p = 0.002$
NVAMD no MA			$p = 0.012$

Table S3. p-values for the paired comparisons of TL in B cells between the groups

Group	NVAMD MA	NVAMD no MA	GA
Controls	$p < 0.001$	$p < 0.001$	$p < 0.001$
NVAMD MA		$p = 0.372$	$p = 0.470$
NVAMD no MA			$p = 0.767$

Table S4. p-values for the paired comparisons of TL in NK cells between the groups

Group	NVAMD MA	NVAMD no MA	GA
Controls	$p = 0.622$	$p = 0.172$	$p = 0.150$
NVAMD MA		$p = 0.498$	$p = 0.672$
NVAMD no MA			$p = 0.747$

Table S5. p-values for the paired comparisons of TL in monocytes between the groups

Group	NVAMD MA	NVAMD no MA	GA
Controls	$p < 0.001$	$p < 0.001$	$p < 0.001$
NVAMD MA		$p = 0.128$	$p = 0.049$
NVAMD no MA			$p = 0.249$

Table S6. p-values for the paired comparisons of TL in all leukocytes between the control group and NVAMD groups

Group	Controls vs NVAMD MA	Controls vs NVAMD no MA	Controls vs GA
T cells	p = 3.9e-07	p = 6.6e-07	p = 0.107
B cells	p = 1.3e-06	p = 4e-04	p = 6.4e-06
NK cells	p = 0.622	p = 0.172	p = 0.15
Monocytes	p = 5.4e-08	p = 7.1e-08	p = 6.3e-07
